# Soluble guanylate cyclase induces cranial vasodilation and headache in adults: a randomized trial.

**DOI:** 10.1093/braincomms/fcaf436

**Published:** 2025-11-05

**Authors:** Nadja B Rasmussen, Casper E Christensen, Håkan Ashina, Faisal M Amin, Rogelio Domínguez-Moreno, Jawdat Abdulla, Jørn Carlsen, Messoud Ashina

**Affiliations:** Department of Neurology, Danish Headache Center, Copenhagen University Hospital—Rigshospitalet, Copenhagen, DK- 2600 Glostrup, Denmark; Department of Clinical Medicine, Faculty of Health and Medical Sciences, University of Copenhagen, DK-2200 Copenhagen, Denmark; Department of Neurology, Danish Headache Center, Copenhagen University Hospital—Rigshospitalet, Copenhagen, DK- 2600 Glostrup, Denmark; Department of Neurology, Danish Headache Center, Copenhagen University Hospital—Rigshospitalet, Copenhagen, DK- 2600 Glostrup, Denmark; Department of Neurology, Danish Headache Center, Copenhagen University Hospital—Rigshospitalet, Copenhagen, DK- 2600 Glostrup, Denmark; Department of Clinical Medicine, Faculty of Health and Medical Sciences, University of Copenhagen, DK-2200 Copenhagen, Denmark; Department of Neurology, Danish Headache Center, Copenhagen University Hospital—Rigshospitalet, Copenhagen, DK- 2600 Glostrup, Denmark; Department of Clinical Medicine, Faculty of Health and Medical Sciences, University of Copenhagen, DK-2200 Copenhagen, Denmark; Difficult-to-Treat Headache Clinic, Department of Neurology and Psychiatry, Instituto Nacional De Ciencias Médicas y Nutrición Salvador Zubirán, 14080 Mexico City, Mexico; Department of Cardiology, Amager-Hvidovre Hospital, Copenhagen, DK-2650 Hvidovre, Denmark; Department of Clinical Medicine, Faculty of Health and Medical Sciences, University of Copenhagen, DK-2200 Copenhagen, Denmark; Department of Cardiology, Copenhagen University Hospital—Rigshospitalet, Copenhagen, DK-2200 Copenhagen, Denmark; Department of Neurology, Danish Headache Center, Copenhagen University Hospital—Rigshospitalet, Copenhagen, DK- 2600 Glostrup, Denmark; Department of Clinical Medicine, Faculty of Health and Medical Sciences, University of Copenhagen, DK-2200 Copenhagen, Denmark

**Keywords:** soluble guanylate cyclase, cyclic guanosine monophosphate, headache, provocation modelling, trigeminovascular system

## Abstract

The role of cranial vasodilation in headache pathogenesis sparks ongoing debate. Soluble guanylate cyclase is an enzyme that catalyses the conversion of guanosine triphosphate to cyclic guanosine monophosphate, leading to relaxation of vascular smooth muscle cells. Activation of this pathway might contribute to headache pathogenesis. We evaluated whether oral ingestion of riociguat, a stimulator of soluble guanylate cyclase, could elicit cranial vasodilation and headache in healthy adults. In this randomized, double-blind, placebo-controlled, two-way crossover study, we enrolled 12 healthy adults (nine females, three males; mean age 27.3 (SD 7.8) years). The participants attended two experimental sessions at a single site in Denmark (Danish Headache Center, Rigshospitalet, Denmark), receiving either a single oral dose riociguat 2.5 mg or placebo. The baseline measurements included the diameter of the superficial temporal artery and the blood flow velocity of the middle cerebral artery. These measurements were repeated over a 240-minute period after ingestion. The participants recorded headache occurrence and associated features in a diary for 12 h post-ingestion. For primary outcome (*n* = 12), results revealed no significant change in the diameter of the superficial temporal artery from baseline to 90 min post-ingestion between riociguat and placebo (*P* = 0.54). However, significant increases in the diameter were observed at 120 min (*P* = 0.02) and 240 min (*P* < 0.01) after riociguat ingestion compared with placebo. The blood flow velocity of the middle cerebral artery decreased significantly from baseline to 240 min post-riociguat ingestion, compared with placebo (*P* < 0.01). Headache was reported by 10 (83%) of 12 participants after riociguat ingestion, compared with three (25%) participants after placebo (*P* = 0.02). In conclusion, oral ingestion of riociguat, a stimulator of soluble guanylate cyclase, induces cranial vasodilation and headache. These findings support the hypothesis that cranial vasodilation mediated via direct soluble guanylate cyclase stimulation contributes to headache pathogenesis. Further research is warranted to delineate the relative contributions of nitric oxide-dependent versus nitric oxide-independent soluble guanylate cyclase activation, and to evaluate guanylate cyclase signalling as a potential therapeutic target in headache disorders.

## Teaser

Rasmussen et al. report that riociguat, a soluble guanylate cyclase stimulator, induces cranial vasodilation and headache in healthy adults. These findings suggest that guanylate cyclase signalling may play a role in headache pathogenesis, highlighting a potential therapeutic target for headache disorders. Further research concerning guanylate cyclase in headache is needed.

## Graphical Abstract

**Graphical Abstract:**
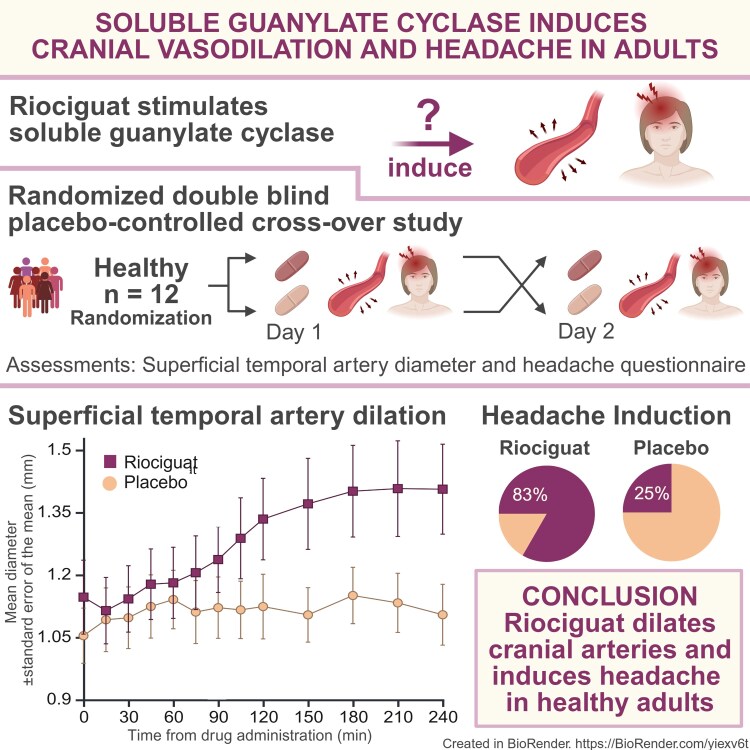

## Introduction

Headache is amongst the most prevalent symptoms known to humans.^1^ The predominant aetiologies include tension-type headache (TTH) and migraine,^1^ although one must not overlook potentially life-threatening secondary causes, such as subarachnoid haemorrhage and meningitis.^2^ Though strides have been made in advancing our understanding of headache pathogenesis,^3^ a lingering question is whether cranial vasodilation plays an important role. It has previously been suggested that cranial vasodilation might represent an epiphenomenon in headache disorders, such as migraine.^4^ However, accumulating evidence indicates that neurovascular mechanisms play a central role in headache pathogenesis.^3^ Recent theories have moved towards a more complex interaction between vascular and neural factors, highlighting the particular importance of initial vessel-to-neuron signalling.^5^ Central to this discussion is soluble guanylate cyclase (sGC), an enzyme that catalyses the intracellular conversion of guanosine triphosphate (GTP) to cyclic guanosine monophosphate (cGMP).^6^ Induction of the cGMP pathway have been shown to promote cranial vasodilation which, in turn, might be implicated in headache pathogenesis.^7-10^

The involvement of cranial vasodilation in headache pathogenesis has evolved from years of human experimental research.^3,10^ These studies involve the administration of potent cranial vasodilators to healthy persons and those with primary headache.^10^ For example, nitric oxide (NO)-donors dilates cranial arteries,^8,9^ induces headache in healthy volunteers,^8,11^ and attacks of primary headaches, such as migraine, tension type headache and cluster headache.^9,12-14^ These findings suggest that while NO is a common mediator in the genesis of head pain, the clinical phenotype that emerges, whether a general headache, migraine, or cluster headache, is likely determined by the individual’s specific underlying susceptibility and pathophysiology.

Within the vascular smooth muscle cells (VSMCs), NO acts primarily through an activation of sGC.^15,16^ Once activated, sGC catalyses GTP to cGMP at an increased rate.^6,17^ The elevation of intracellular cGMP facilitates a cascade of intracellular events initiated by an increase in protein kinase G (PKG), phosphodiesterase’s (PDEs) and cyclic nucleotide-gated (CNG) ion channels. This ultimately results in decreased intracellular calcium levels causing vasodilation, alongside a simultaneous efflux of potassium ions into the perivascular space.^18^ This is hypothesized to provide both mechanical and chemical stimuli that concurrently activate and sensitize primary sensory afferents, initiating ascending nociceptive messaging.^5^

Here, we report the results of a randomized, double-blind, placebo-controlled, two-way crossover study that evaluated the incidence of cranial vasodilation (with change in diameter of superficial temporal artery from baseline to 90 min as primary outcome) and headache after oral ingestion of riociguat, a sGC stimulator, in healthy adults.

## Materials and methods

The study protocol was approved by the Regional Health Research Ethics Committee of the Capital Region of Denmark (H-22020347) and registered with ClinicalTrials.gov (Identifier: NCT05582811). The study was conducted in accordance with the Declaration of Helsinki, with later revisions. All participants provided written informed consent prior to their study inclusion. The study was conducted from October 2022 to June 2023. The lead author (NBR) affirms that the manuscript is an honest, accurate, and transparent account of the study being reported; and that no important aspects of the study have been omitted.

### Design

This study applied a randomized, double-blind, placebo-controlled, two-way crossover design involving healthy adults. It was conducted at a single site in Denmark (Danish Headache Center, Rigshospitalet, Denmark). The participants attended an initial screening visit, during which eligibility was determined based on predefined inclusion and exclusion criteria. Eligible participants were then scheduled for two separate experiment days, during which they received either riociguat or placebo in a randomized order (Supplementary Figs 1 and 2).

### Participants

The participants were recruited through advertisements placed at universities and hospitals, as well as via online advertisements on https://www.forsoegsperson.dk/, a digital platform dedicated to the recruitment of study participants.

The inclusion criteria required participants to be healthy adults aged 18 to 45 years, with no history of headache disorders (except for infrequent episodic TTH), cardiovascular diseases, or any other major illnesses. Furthermore, eligible participants had to report a body weight between 50 and 100 kg, be non-smokers, and report no family history of severe cardiovascular disease. A complete list of the inclusion and exclusion criteria is available at ClinicalTrials.gov (Identifier: NCT05582811).

### Randomization

The experimental drug, riociguat (Adempas®), produced by Bayer, was available in different oral tablet doses. A single 2.5 mg oral dose of riociguat was selected for the study based on data from our pilot experiment, where we tested doses 2.5 and 5 mg (data not shown).^19^ One healthy adult completed the pilot dose-finding study. Both 2.5 mg and 5 mg doses of riociguat, induced STA dilation and moderate to severe headache, confirming an induction effect at the lower dose. An unfavourable number of adverse effects were seen with the higher compared to the lower dose of riociguat (data not shown). Placebo tablets were composed of lactose monohydrate, potato starch, gelatine A, purified water, magnesium stearate MF2V, and talc. For blinding purposes, both riociguat and placebo tablets were identically encapsulated. Randomization and allocation concealment were managed by independent pharmacy staff to maintain blinding. A balanced 1:1 randomization was applied to ensure an equal number of participants in both the riociguat group and placebo group. The randomization code was securely stored in sealed and opaque envelopes and remained inaccessible to the investigators until the study was completed. Participants were assigned to a specific study ID, with two corresponding containers holding either a capsule with riociguat or placebo, respectively, of identical appearance for the two experimental days.

### Screening visit

At the screening visit, we gathered detailed medical and psychiatric histories, which included access to medical journals and previous/current use of prescription medication. We assessed any potential exclusion criteria, including any current or previous known primary or secondary headache disorder(s) apart from tension type headache ≤ 1 day per month and any known history of first-line relative with migraine. The participants underwent a standard physical examination, including neurological status, vital signs recording, blood sampling, and a 12-lead electrocardiogram (ECG). No standardized questionnaires were used to evaluate psychiatric disease. A urine pregnancy test was also performed in women of childbearing potential.

### Experiment days

Each participant attended two separate experiment days, with a minimum of five days serving as a washout period in between. The participants were mandated to adhere to a fasting period of 10 h and confirm a headache-free period of at least 48 h prior to each experiment day. The study room was the same for all experiment days and was controlled for temperature, noise and light.

Upon arrival, a urine pregnancy test was repeated in women of childbearing potential on both experiment days prior to further assessments. A peripheral venous catheter was carefully inserted into a vein in the antecubital area, providing intravenous access for the duration of the in-hospital phase. The participants were instructed to rest in the supine position for a period of 30 min prior to baseline measurements, which encompassed vital signs, a 12-lead ECG, diameter of the superficial temporal artery (STA), and blood flow velocity of the middle cerebral artery (V_MCA_). Concurrently to baseline measurements participants confirmed absence of headache and associated symptoms. The participants were then administered either an oral capsule containing riociguat or placebo. Throughout the ensuing 240-minute in-hospital experiment phase, vascular measurements and headache assessments were registered repeatedly at fixed timepoints (Supplementary Figs 1 and 2). Upon completion of the in-hospital phase, a 12-lead ECG was performed once more in a supine position. The participants were then discharged and instructed to complete a headache diary with hourly entries for the remaining time of the 12-hours period after ingestion of riociguat or placebo. Of note, participants were permitted to treat any induced headache with over-the-counter rescue medication at any time.

### Diameter of the superficial temporal artery

The STA diameter was measured using a high-resolution ultrasound scanner (Dermascan C unit, 20 MHz, Cortex Technology, Hadsund, Denmark).^20^ The probe was positioned on the skin surface, capturing a transverse section of the frontal branch of the STA. To ensure consistency, measurements were conducted on the same side (either left or right) for each participant across both experiment days, and all measurements were performed by the same trained personnel member. An average of four separate measurements were recorded for STA diameter at each time point forming the basis for calculating a corresponding mean STA value. Furthermore, these measurements were conducted at regular intervals throughout the in-hospital phase, every 15 min from 0 to 120 min and every 30 min from 120 to 240 min, after the ingestion of either riociguat or placebo.

### Blood flow velocity of the middle cerebral artery

Bilateral V_MCA_ measurements were performed using transcranial Doppler (TCD, 2 MHz handheld ultrasound probes, DWL). A mean V_MCA_ value was derived from an average of up to four (intended) measurements recorded on each side. Concurrently, end-tidal CO2 pressure was recorded using a nasal catheter devoid of respiratory resistance (Standard Adult Nasale Cannula, straight prongs, Intersurgical Ltd, UK). These V_MCA_ measurements were carried out during the in-hospital phase at consistent intervals: every 15 min from 0 to 120 min, and subsequently every 30 min from 120 to 240 min, post-ingestion of riociguat or placebo.

### Vital signs

Throughout the 240-minute in-hospital experiment phase, heart rate (HR) and blood pressure were monitored at regular intervals using a bedside monitor. Measurements were taken every 10 min from 0 to 120 min post-ingestion, and then every 30 min from 120 to 240 min.

### Headache and associated symptoms

A standardized headache questionnaire and at-home diary was used to record headache features, associated symptoms, rescue medication use, and adverse events during the 12-hour observation period post-ingestion of either riociguat or placebo (Supplementary Fig. 2B). They were designed to rate the headache intensity on an 11-point numeric rating scale where 0 signifies ‘no pain’, 1 represents ‘changed sensation in the head’ and 10 represents ‘worst imaginable headache’. Furthermore, the diary included additional queries to ascertain the occurrence of unusual tiredness, neck stiffness, yawning, mood swings, trouble concentrating, hunger, and thirst.

### Statistical analysis

The sample size calculations were performed with a paired *t*-test, assuming a 51% difference in STA diameter between the riociguat and placebo groups and standard variation less than 20.1%. To achieve a power of 90% at a two-sided significance level of 5%, a sample size of five participants were deemed necessary. However, to improve the accuracy and reliability of our data, the enrolment was expanded to include 12 participants. In the event of participant withdrawal prior to the completion of the study, replacements were made. The final analysis included only those participants who had successfully completed both days of the experiment.

Demographic and clinical characteristics were summarized using descriptive statistics, as appropriate. Continuous data were presented as means with standard deviations (SD) for normally distributed data and as medians with ranges for non-normally distributed data. Categorical data were presented as absolute numbers and percentages.

The primary outcome was the change in STA diameter from baseline to 90 min after riociguat ingestion, compared with placebo. The secondary outcomes were (I) the change in V_MCA_ from baseline to 240 min post-riociguat ingestion, (II) the change in HR from baseline to 240 min post-riociguat ingestion, and (III) the incidence of headache from baseline to 12 h post-riociguat ingestion, all in comparison with placebo. The predefined exploratory outcomes were (i) the severity of headache, if present, using an 11-point numerical rating scale ranging from 0 (‘no pain’) to 10 (‘worst pain imaginable’) from baseline to 12 h post-riociguat ingestion, (ii) headache characteristics during the same timeframe, (iii) use of rescue medication from baseline to 12 h post-riociguat ingestion and (iiii) the change in STA diameter from baseline to 4 h after riociguat-ingestion, compared with placebo.

A linear mixed model (LMM) was used to analyse changes in STA diameter at 90, 120 (post-hoc analysis) and 240 min from baseline, as well as changes in V_MCA_ at 240 min from baseline. Time and intervention were included as fixed effects and participants as a random effect. To address any potential autocorrelation issues, we conducted sensitivity analyses for all LMM analyses. Specifically, we fitted the models with a first-order continuous autoregressive process and included the individual participant as a random effect for each study day, setting the correlation parameter to 0.5. Normality of residuals was tested using visual inspection of qqplots. Differences in mean HR and mean arterial pressure (MAP) from baseline to 4 h, as well as headache intensity from baseline to 12 h post-riociguat ingestion, were analysed using the Wilcoxon signed-rank test based on the area under the curve (AUC), calculated with the trapezium rule.

The incidence of headache was analysed as paired binary categorical data using McNemar’s test with continuity correction. Post-hoc analysis with student *t*-test was performed to compare baseline values for mean HR, MAP, mean STA diameter, and mean V_MCA_ between the two experiment days. We tested for significance at a 5% level for all comparisons. All statistical analyses were performed using R (version 4.4.1) or GraphPad Prism 10.3.0.

## Results

### Participants

A total of 14 healthy adults were enrolled and underwent randomization (Supplementary Fig. 2). Two participants withdrew after the first experiment day. Thus, the final analysis included 12 healthy adults, comprising nine women and three men, with a mean age of 27.3 (SD 7.8) years. Baseline characteristics of the study population are outlined in Table 1. Post-hoc analysis revealed no differences in baseline values for mean HR, MAP, STA diameter, and V_MCA_ between the two experiment days (Supplementary Table 1).

**Table 1:** Screening demographic and clinical characteristics of the study population

Characteristics	Healthy volunteers (*n* = 12)
Age, years, mean (SD)	27.3 (7.8)
Sex, *n* (%)	
Male	3 (25)
Female	9 (75)
Weight, kg, mean (SD)	72.4 (12.7)
Height, m, mean (SD)	1.76 (0.1)
Body Mass Index, mean (SD)	23.4 (2.1)
Race	
White, *n* (%)	12 (100)
Blood pressure, systolic, mmHg, mean (SD)	123,9 (11.2)
Blood pressure, diastolic, mmHg, mean (SD)	79,8 (7.8)
Mean Arterial Pressure, calculated, mean (SD)	94,5 (7.5)
Heart rate, beats/minute, mean (SD)	68.4 (15.4)

SD; standard deviation.

Demographic and clinical characteristics collected at the screening visit.

### Vascular and haemodynamic outcomes

**Figure 1:**
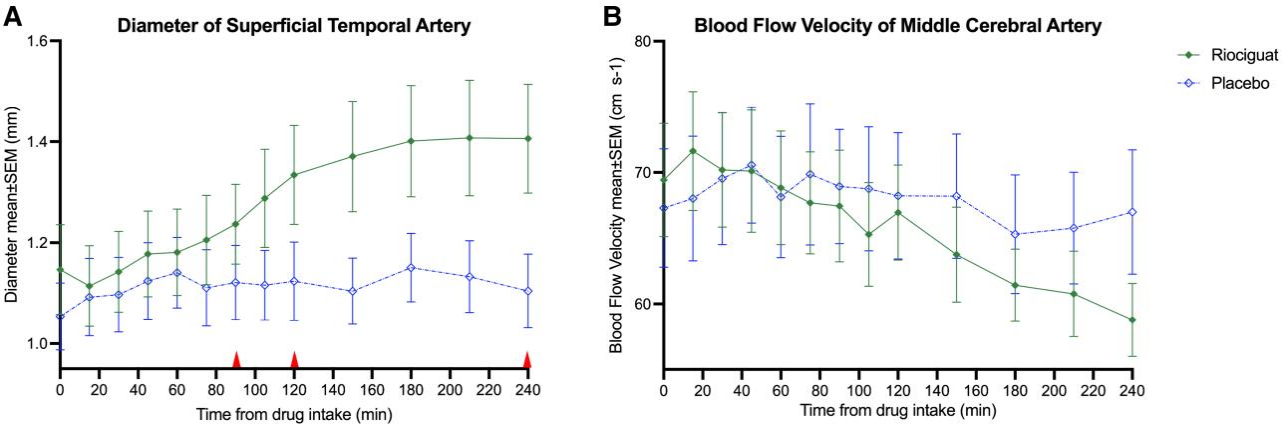
**Superficial temporal artery diameter and middle cerebral artery blood flow velocity.** The figure shows mean ± SEM (standard error of the mean) of (**A**) superficial temporal artery diameter (mm) measurements and (**B**) middle cerebral artery blood flow velocity (V_MCA_) (cm s^−1^) measurements after intake of riociguat (green) and placebo (blue) during the in-hospital phase on experiment days from baseline until 240 min (minutes). In (**A**) Diameter of superficial temporal artery, specific timepoints for outcomes are marked with red arrows on the x-axis (primary outcome timepoint 90 min (*P* = 0.54), post hoc exploratory outcome timepoint 120 min (*P* = 0.02), and pre-specified exploratory outcome timepoint 240 min (*P* < 0.01)). A linear mixed model (LMM) was used to analyse changes in STA diameter at 90, 120 (post-hoc analysis) and 240 min from baseline, as well as changes in V_MCA_ at 240 min from baseline, and included a sample size of 12 healthy adults (*N* = 12). Created in BioRender. Rasmussen, **N**. (2025) https://BioRender.com/2xuqf2f.

No significant difference in STA diameter was observed between riociguat and placebo from baseline to 90 min post-ingestion (*P* = 0.54). However, post-hoc analysis demonstrated a significant increase in STA diameter after riociguat compared with placebo at 120 min from baseline (*P* = 0.02) and for exploratory outcome 240 min from baseline (*P* < 0.01; Fig. 1). A decrease in V_MCA_ was noted from baseline to 240 min post-ingestion of riociguat, as compared with placebo (*P* < 0.01; Fig. 1). Furthermore, administration of riociguat was associated with an increase in AUC for HR from baseline to 240 min, compared to placebo (*P* < 0.01). However, no significant differences were found in AUC for MAP during the same period (*P* = 0.062; Supplementary Fig. 3).

### Headache incidence and characteristics

**Figure 2:**
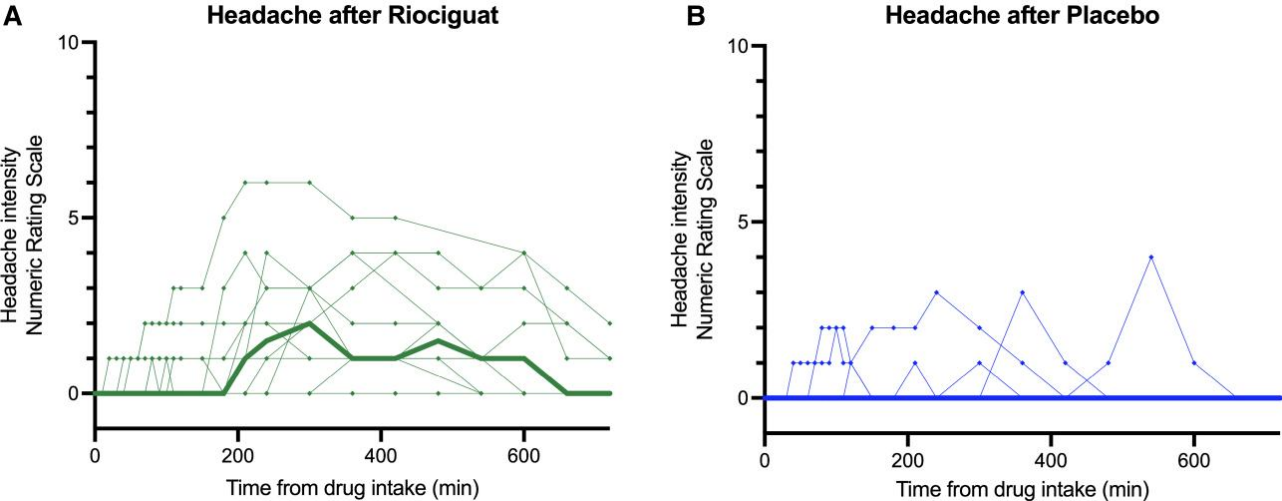
**Headache after riociguat and placebo.** The figure displays headache intensity after (**A**) riociguat (green) and (**B**) placebo (blue) on experiment days. Ten of 12 (83%) participants reported headache after riociguat, compared to 3/12 (25%) participants reporting headache after placebo (*P* = 0.02). Median (thick line) and individual (thin lines, dots represent individual intensity rating at a given timepoint) headache intensity on a numerical rating scale (NRS) from 0–10 from baseline until 12 h. Wilcoxon signed-rank test based on the area under the curve (AUC), calculated with the trapezium rule, was used for statistical analysis. The headache intensity scores, quantified as AUC values, was significantly higher after riociguat, as compared with placebo (*P* < 0.01). For those experiencing headache, median peak headache intensity score was higher following riociguat (2.5; range, 0 to 6) than after placebo (0; range, 0 to 4; *P* = 0.01). Created in BioRender. Rasmussen, N. (2025) https://BioRender.com/vcmmbx8.

During the 12-hour observational period, ten (83%) of 12 participants experienced headache after ingestion of riociguat, compared with three (25%) after placebo (*P* = 0.02). The headache intensity scores, quantified as AUC values, were significantly higher after riociguat, as compared with placebo (*P* < 0.01; Fig. 2). For those experiencing headache, median peak headache intensity score was higher following riociguat (2.5; range, 0 to 6) than after placebo (0; range, 0 to 4; *P* = 0.01) (Fig. 2). Median time to headache start was 90 min following riociguat and 70 min for placebo. Moreover, eight participants used simple analgesics (i.e. ibuprofen and paracetamol) to treat their headache, and this was exclusively after riociguat. A detailed overview of headache characteristics and analgesic use is provided in Table 2.

**Table 2:** Clinical characteristics of headache and associated symptoms in healthy adults after intake of riociguat and placebo (0–12 h observation period)

ID number Treatment	Peak headache (duration), minutes	Headache characteristics^a^	Associated symptoms^b^	Migraine-like^c^	Treatment for headache
1					
Riociguat	210 (340)	Bilat/2/pres/+	−/−/−	No	Paracetamol
Placebo	None	—	—	—	—
2					
Riociguat	240 (580)	Bilat/4/pres/−	−/+/−	No	Paracetamol, Ibuprofen
Placebo	None	—	—	—	—
3					
Riociguat	210 (600)	Bilat/4/pres + throb/+	−/−/−	No	Paracetamol
Placebo	360 (150)	Bilat/3/pres/−	−/−/−	No	None
4					
Riociguat	80 (420)	Bilat/1/pres/−	−/−/−	No	None
Placebo	None	—	—	—	—
5					
Riociguat	210 (610)	Bilat/6/pres/+	+/+/−	Yes	Paracetamol, Ibuprofen
Placebo	240 (350)	Bilat/3/pres/+	−/+/−	No	None
6					
Riociguat	None	—	—	—	—
Placebo	None	—	—	—	—
7					
Riociguat	None	—	—	—	—
Placebo	None	—	—	—	—
8					
Riociguat	210 (440)	Bilat/2/pres/+	+/−/−	No	Paracetamol
Placebo	None	—	—	—	—
9					
Riociguat	360 (300)	Bilat/1/pres/−	−/−/−	No	None
Placebo	None	—	—	—	—
10					
Riociguat	420 (540)	Bilat/4/pres/+	+/−/−	Yes	Paracetamol
Placebo	540 (380)	Bilat/4/pres/−	+/+/−	No	None
11					
Riociguat	360 (560)	Bilat/4/pres + throb/+	+/−/−	Yes	Paracetamol
Placebo	None	—	—	—	—
12					
Riociguat	300 (550)	Bilat/3/throb/+	−/−/−	No	Paracetamol
Placebo	None	—	—	—	—

^a^Localization/intensity/quality (bilat = bilateral; unilat = unilateral/pres = pressing; throb = throbbing/aggravation (by cough during in-hospital phase (0–4 h after intake of riociguat or placebo) and by movement during out-hospital phase (5–12 h after intake of riociguat or placebo)).

^b^Nausea/photophobia/phonophobia.

^c^Migraine-like are defined according to fulfilling C and D criteria in ICHD-3 classification of migraine without aura.

### Adverse effects

Facial flushing, recorded both during the in-hospital phase and post-discharge, was recorded for 11 (92%) participants after riociguat and three (25%) participants after placebo. Notably, post-discharge, four participants self-reported facial flushing after ingestion of riociguat, with no reports post-placebo. A summary of registered adverse effects is provided in Supplementary Table 2.

## Discussion

In this randomized, double-blind, placebo-controlled, two-way crossover study, we found that oral ingestion of riociguat, a sGC stimulator, induced cranial vasodilation and headache in healthy adults. Notably, riociguat did not cause STA vasodilation at 90 min as hypothesized. It did however cause a STA dilation after 120- and 240-minutes post-ingestion and a V_MCA_ decrease after 240 min, indicative of cerebral vasodilation. Furthermore, 10 (83%) of 12 participants reported experiencing headache following riociguat ingestion, necessitating the use of simple analgesics for pain management in most of the cases.

**Figure 3:**
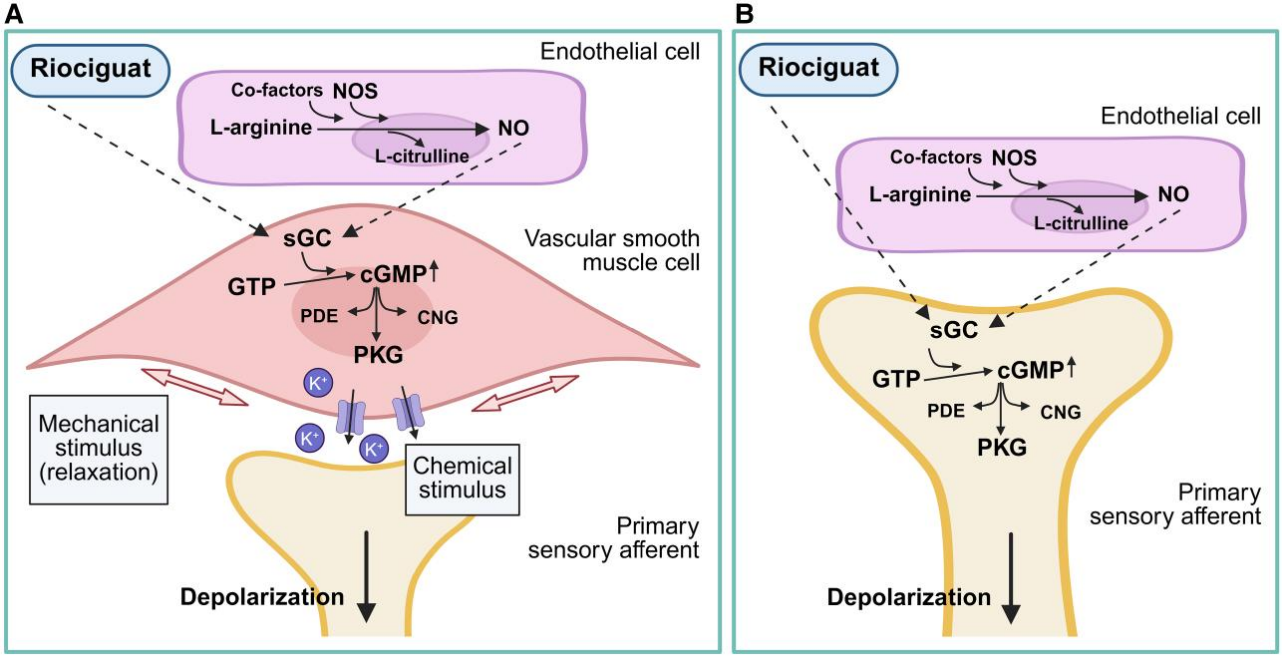
**Riociguat in the NO-sGC-cGMP pathway in the vasculature and sensory afferents.** cGMP, cyclic guanosine monophosphate; CNG, cyclic nucleotide-gated ion channels; GTP guanosine triphosphate; K^+^, potassium; NO, nitric oxide; NOS, nitric oxide synthase; PDE, phosphodiesterase; PKG, protein kinase G; sGC, soluble guanylate cyclase. The figure illustrates two hypothesized mechanisms for riociguat induced headache. (**A**) Illustrates the vessel-to-neuron hypothesis, with primary source of NO from endothelial cells and (**B**) illustrates the direct activation of sensory neuron hypothesis. Created in BioRender. Rasmussen, N. (2025) https://BioRender.com/neh5ef1.

Our findings suggest that activation of sGC elevates intracellular cGMP, promotes cranial artery dilation, and contributes to headache induction. This is consistent with previous research demonstrating that administration of NO donors, such as nitroglycerin, induce cranial vasodilation,^9^ headache in healthy volunteers,^11^ and attacks of primary headaches such as tension-type headache,^12^ cluster headache^13^ and particularly migraine.^9,14^ The present results extend these observations by directly stimulating sGC with riociguat, bypassing upstream determinators of NO formation (Fig. 3)^15,16,21^ We thereby confirm that activation of this enzyme is sufficient to induce cranial vasodilation and headache.

The delayed onset of STA dilation observed in our study, with significant changes occurring at 120- and 240-minutes post-riociguat ingestion, aligns with the pharmacokinetic profile of riociguat.^22-24^ Riociguat reaches peak plasma concentrations within 1.5 h after oral administration^19,22-24^ and its vasodilatory effects might become more pronounced as plasma levels increase. The time course of headache onset and cranial vasodilation suggests that these vascular changes are temporally associated with the development of headache, supporting a possible causal relationship. This temporal association reinforces the concept that vascular mechanisms might contribute to headache pathogenesis, although it does not exclude the involvement of other mechanisms.

### Implications for the trigeminovascular system

Our results have important implications for understanding the mechanisms underlying headache disorders, particularly TTH, cluster headache and migraine. The trigeminovascular system, which comprises the trigeminal nerve and its axonal projections to the cranial vasculature, plays a central role in headache pathogenesis.^3^ Activation of perivascular nociceptors by mechanical and chemical stimuli resulting from vasodilation is hypothesized to initiate nociceptive signalling to the CNS, leading to the perception of headache.^5^ By demonstrating that sGC stimulation induces both cranial vasodilation and headache, our study provides evidence that this signalling pathway might be a critical mediator in the trigeminovascular activation underlying headache. Although we recognize that vasodilation could potentially result from the activation of the trigeminovascular reflex secondary to headache, our data indicate that the median onset of headache following riociguat administration is delayed relative to the onset of STA vasodilation. This timing suggests that vasodilation is more likely an initiating event rather than a consequence of headache. We agree that further research is needed to fully elucidate the interrelationship between these phenomena.

The observation that riociguat induced headache in healthy adults without individual history of headache disorders (except for infrequent episodic TTH) suggests that sGC activation can elicit headache even in the absence of underlying susceptibility. This finding underscores the potential of sGC as a universal mediator of headache and highlights its relevance as a therapeutic target. It also raises the possibility that people with heightened sensitivity to sGC might be more prone to develop headache.

### Hypothesized direct cellular effects of soluble guanylate cyclase activation in migraine pathogenesis

Evidence suggests that riociguat has limited penetration across the blood-brain-barrier, and thus poor access to the CNS.^25,26^ In our view, this finding supports a primarily peripheral site of action, possibly involving meningeal vasodilation and headache induction. However, we cannot entirely exclude a potential CNS effect.

One proposed effect involves sGC-mediated cGMP accumulation in VSMCs within the walls of meningeal arteries. This increase in cGMP prompts several downstream events that reduce intracellular calcium levels.^27^ Among these events is the activation of protein kinase G, which phosphorylates targets that lower cytosolic calcium and promote the opening of ATP-sensitive potassium (K_ATP_) channels and large conductance calcium-activated potassium (BK_Ca_) channels.^18,28,29^ As potassium ions efflux from VSMCs, the cells hyperpolarize, causing relaxation and vasodilation of meningeal arteries.^28^ Such vasodilation might stretch perivascular meningeal nociceptors, causing mechanical activation.^5^ Furthermore, increased potassium concentration in the perivascular space might provide chemical stimuli to further sensitize these nociceptors.^5^ These processes together are hypothesized to convert vascular responses into ascending nociceptive signalling, ultimately yielding the perception of headache.^5^

A second hypothesis posits that sGC-generated cGMP can act directly in nociceptive neurons, independent of its vascular effects. While this pathway is less characterized, there is evidence that cGMP upregulation can modulate meningeal nociceptor activity in rodents.^30^ This modulation might increase the responsiveness of nociceptive neurons, priming them to fire action potentials in response to otherwise subthreshold stimuli. As a result, cGMP-dependent neuronal changes could alone initiate headache without requiring pronounced vascular responses. Further research is, nevertheless, needed to ascertain whether sGC stimulation in meningeal nociceptors facilitate their signalling.

Emerging studies highlight a third consideration. Satellite glial cells in peripheral ganglia appear crucial to pain regulation.^31^ Recent preclinical work indicates that sGC activation in satellite glial cells can potentiate neuronal excitability in both dorsal root and trigeminal ganglia, raising the possibility that riociguat-mediated stimulation of these cells could promote headache pathogenesis.^32^

### Haemodynamic responses

The observation that riociguat induced significant vasodilation and an increased heart rate while maintaining stable mean arterial pressure is consistent with established compensatory cardiovascular mechanisms, such as reflex tachycardia and vascular autoregulation. These findings suggest that the induction of headache is more likely attributable to direct cranial vascular alterations rather than systemic hypotension. It is important to note that these conclusions are derived from acute experimental data, and further research is needed to elucidate the underlying compensatory mechanisms and their long-term implications in the context of sGC-targeted therapies.

### Therapeutic implications

Our findings open new avenues for exploring sGC as a therapeutic target in headache disorders. Inhibitors or partial modulators of this enzyme could possibly prevent cranial vasodilation and alleviate head pain. Moreover, sGC exists in two isoforms—α1β1 and α2β1—with different tissue distributions,^33,34^ presenting an opportunity to explore isoform-specific modulation to further assess the enzyme’s involvement in headache pathogenesis and develop tailored treatments. Although isoform-specific modulators are not currently available, the development of such agents might pave the way for targeted therapies that minimize side effects.

### Limitations

This study has several limitations that warrant consideration. First, the use of a single dose precludes us from assessing dose-response relationships. Different doses of riociguat might produce varying effects on cranial vasodilation and headache induction. In particular, higher doses of riociguat could enhance the rate of headache induction, although potentially also lead to simultaneous more pronounced cranial and systemic vascular effects, thereby increasing the risk of adverse events, including potential serious adverse events. Based on recommendations from a specialized cardiologist, the observed adverse events from our pilot study, and the present outcome measures, we determined that the 2.5 mg dose was optimal for this study. Although it remains uncertain whether a lower dose would also be sufficient, our findings indicate that the 2.5 mg dose effectively induces cranial vasodilation and headache with an acceptable safety profile. Preclinical dose-ranging studies might provide valuable information on the potential threshold levels required to induce these effects. However, our results are comparable to those observed in previous studies using nitric oxide donors and sildenafil.^8,11,14,35^ Second, certain symptoms and physiological changes in participants—such as facial flushing and heart rate increases—can compromise blinding to some extent. These expected effects of riociguat could lead to confirmation and observer bias, as both participants and assessors might have inferred the drug allocation. However, complete blinding is unfeasible under these circumstances. Lastly, our study population consisted of healthy adults, predominantly female, which may limit the generalizability of our findings to other groups, such as males and individuals with headache disorders. Further research is warranted to determine whether sGC exerts similar effects in these populations.

## Conclusions

Riociguat, a sGC stimulator, elicits cranial vasodilation and headache in healthy adults. These findings support the notion that sGC-mediated vasodilation might play a causal role in headache pathogenesis. Thus, targeting sGC signalling represents a promising therapeutic avenue for the treatment of headache disorders. Further research is warranted to explore this signalling pathway in experimental studies and to develop targeted therapies.

## Supplementary Material

fcaf436_Supplementary_Data

## Data Availability

Codes generated for the purpose of statistical analyses in R and GraphPad Prism are included in Supplementary materials. Upon reasonable request, the corresponding author will provide the necessary data and materials to interested researchers for the purpose of academic scrutiny, reproducibility, and further scientific investigation.
