# Supplementary material for: Soluble guanylate cyclase induces cranial vasodilation and headache in adults: a randomized trial
Source: Brain Commun. 2025 Nov 5;7(6):fcaf436. doi: 10.1093/braincomms/fcaf436 (PMC12663088; doi:10.1093/braincomms/fcaf436)

**Supplementary material**

**Supplementary Table 1.** Baseline vascular values on experiment days before administration of study drug.

|  |  | Riociguat | Placebo | *p*-value^a^ |
| --- | --- | --- | --- | --- |
| STA, mm, mean (SD) | | 1.146 ±0.31 | 1.054 ±0.23 | 0.302 |
| V_MCA_, cm/s, mean (SD) | | 69.4 ±15.0 | 67.3 ±15.6 | 0.373 |
| HR, beats/min, mean (SD) | | 62.8 ±10.1 | 61.7 ±11.8 | 0.363 |
| MAP, mmHg, mean (SD) | | 77.3 ±5.7 | 77.6 ±6.8 | 0.821 |

^a^student t-test

Baseline values of superficial temporal artery (STA), middle cerebral artery blood flow velocity (V_MCA_), heart rate (HR) and mean arterial pressure (MAP) (calculated) prior to study drug administration on the two experiment days. Post hoc analysis revealed no significant difference in vascular parameters prior to administration of riociguat compared to placebo.

**Supplementary Table 2.** Adverse Effects of Riociguat and Placebo

| 0-12 hours | Riociguat | Placebo |  |
| --- | --- | --- | --- |
| Unusual tiredness | 5 | 4 |  |
| Neck stiffness | 0 | 0 |  |
| Yawning | 1 | 0 |  |
| Mood swings | 1 | 0 |  |
| Difficulty concentrating | 4 | 1 |  |
| Hunger | 6 | 3 |  |
| Thirst | 2 | 1 |  |
| Flushing (observed) /Redness (self-reported) | 11/4 | 3/0 |  |
| Heat sensation (face/body) | 5 | 0 |  |
| Heart palpitations | 2 | 0 |  |
| Other | 6 | 1 |  |
| - *Dizziness* | 3 | 0 |  |
| - *Rhinorrhea* | 1 | 0 |  |
| - *Nasal congestion* | 4 | 0 |  |
| - *Feeling cold* | 1 | 1 |  |
| - *Feeling unwell* | 1 | 0 |  |
| - *Cold sweats* | 1 | 0 |  |
| - *Warm sensation at intravenous access* | 2 | 0 |  |
| - *Sore lower back* | 1 | 1 |  |
| - *Eye watering* | 2 | 0 |  |
| - *Conjunctival injection* | 1 | 0 |  |
| - *Dry mouth/throat* | 1 | 0 |  |
| - *Presyncope* | 1 | 0 |  |
| - *Soreness to eye movements* | 1 | 0 |  |
| - *Feeling uneasy in upper stomach region* | 1 | 0 |  |
| Total | **43** | **13** |  |

Listed adverse effects of riociguat and placebo registered with pre-specified questions in the headache questionnaire and headache diary in the 12-hour period after administration of the study drug. The option ‘Other’ allowed the participant to describe anything other than the pre-specified questions.

**Supplementary Figure 1.** CONSORT 2010 Study Flow Diagram


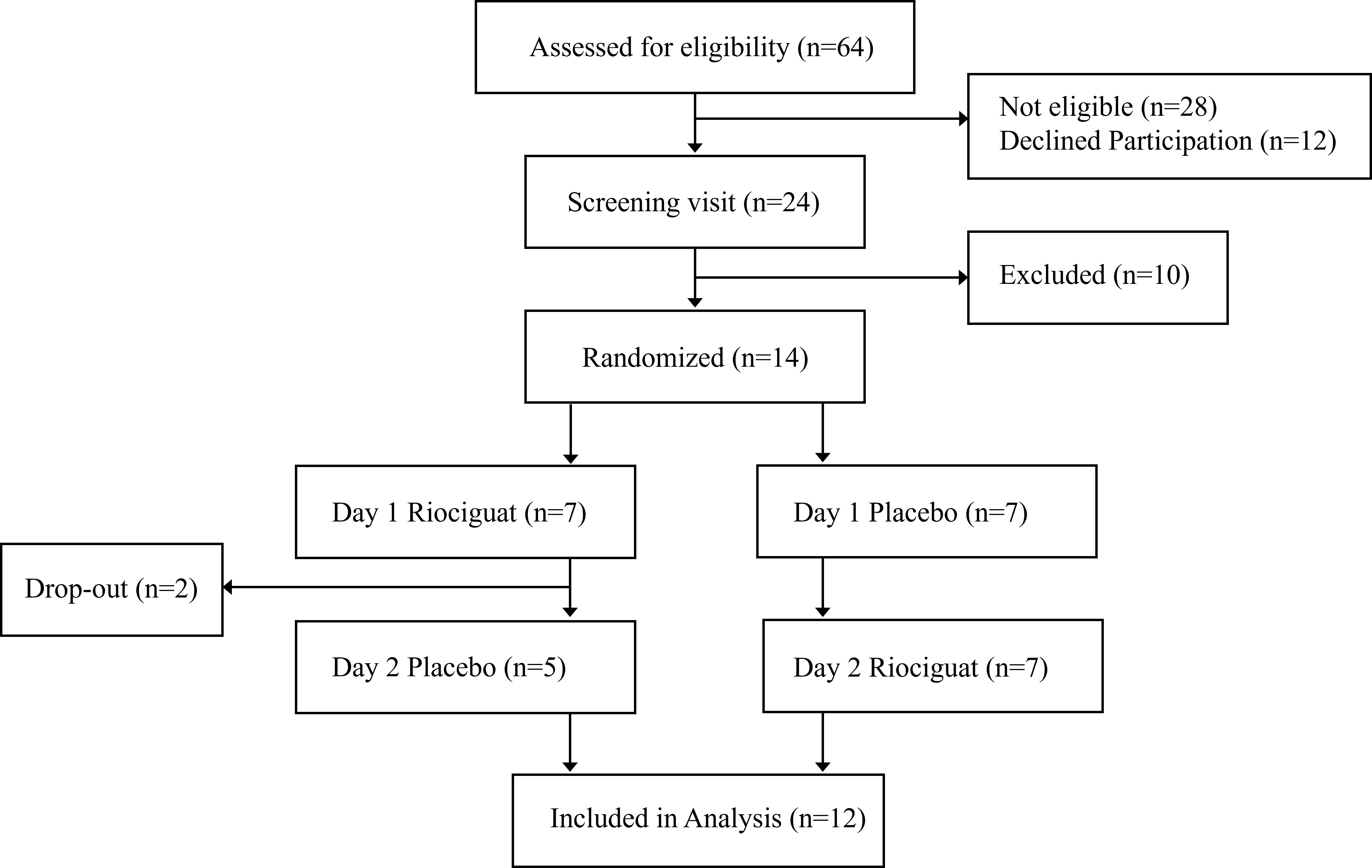


A total of 64 volunteers were contacted and assessed for eligibility, of which 28 were not eligible and 12 did not wish to participate. Thus 24 volunteers were invited for a screening visit, where they were informed about the study, and all chose to sign the informed consent form. Thereafter they were all assessed for inclusion and exclusion criteria, and 10 were excluded. The remaining 14 were included and randomized at the first experiment day. Two subjects dropped out of the study prior to the second experiment day, and a total of 12 participants completed both experiment days and were included in the final analysis.

**Supplementary Figure 2. Study design overview**


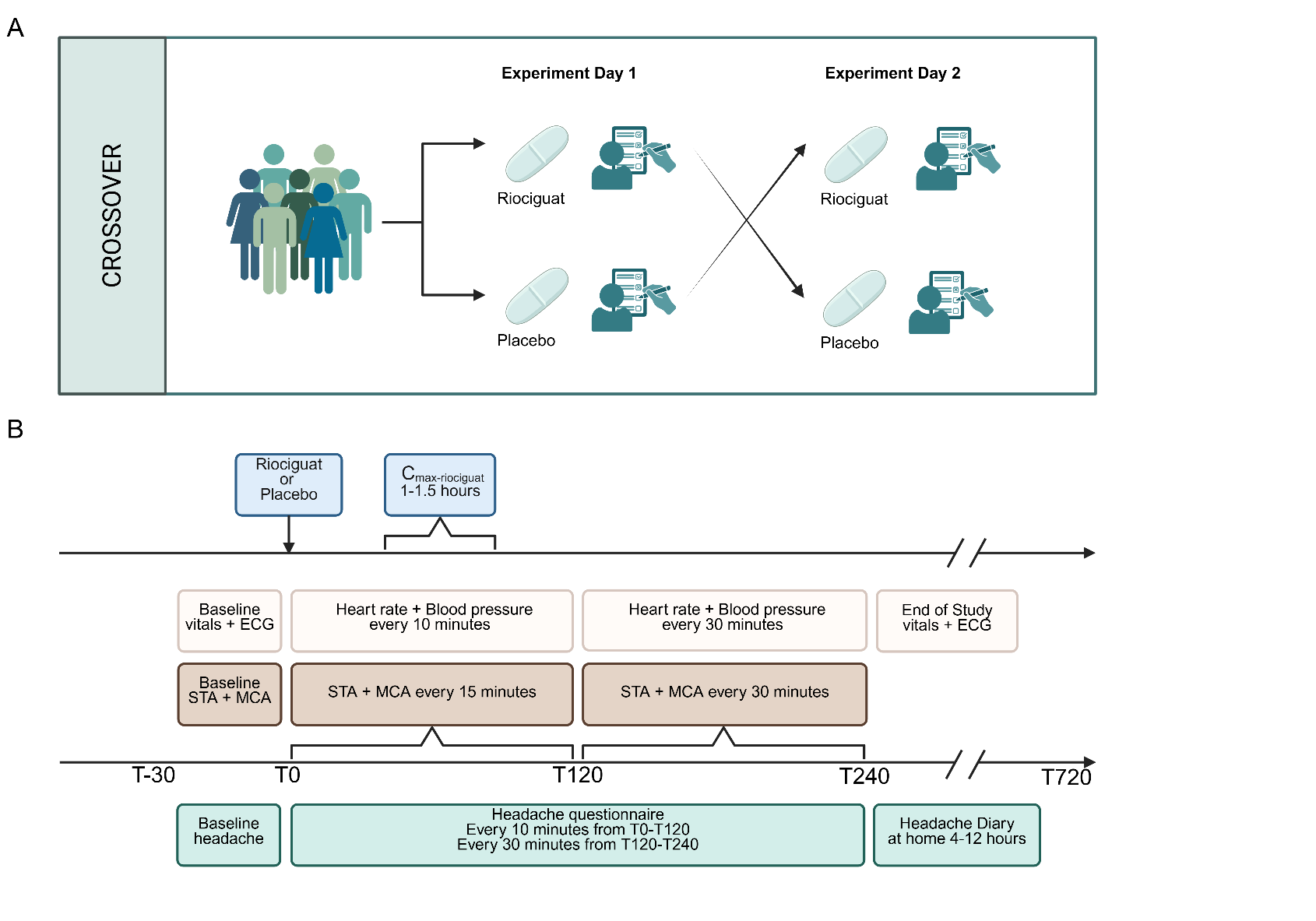


Overview of A) overall study crossover design and B) flow chart of measurements on experiment days.

Cmax-riociguat, expected maximum plasma concentration of riociguat; MCA, middle cerebral artery; T-30, 30 minutes prior to baseline; T0, baseline; T120, timepoint 120 minutes after baseline; T240, timepoint 240 minutes after baseline; T720, timepoint 720 minutes after baseline.

Created in BioRender. Rasmussen, N. (2025) https://BioRender.com/y2kp4xv

**Supplementary Figure 3. Heart Rate and Mean Arterial Pressure after Riociguat and Placebo.**

**
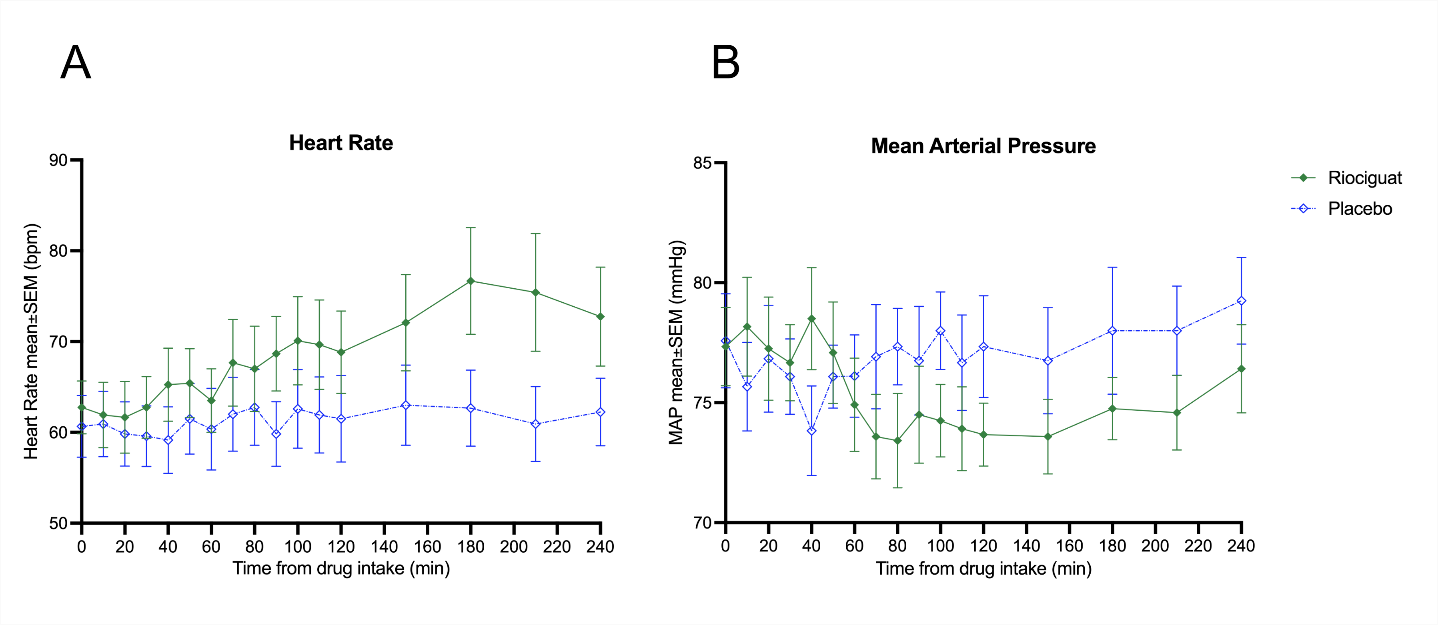
**

The figure shows mean±SEM (standard error of the mean) of A) Heart Rate (HR) (beats/min, bpm) and B) Mean Arterial Pressure (MAP) (mmHg) on experiment days after intake of riociguat (green) compared to placebo (blue) from baseline until 4 hours. Differences in mean HR and MAP from baseline to 4 hours were analyzed using the Wilcoxon signed-rank test based on the area under the curve (AUC), calculated with the trapezium rule, and included a sample size of 12 healthy adults (N=12). Riociguat was associated with an increase in AUC for HR from baseline to 240 minutes, compared to placebo (P < 0.01). However, no significant differences were found in AUC for MAP during the same period (P = 0.062).

Created in BioRender. Rasmussen, N. (2025) https://BioRender.com/dr3v9pu

**Supplementry material for statistical analysis**

Codes applied in R for statistical analysis of STA data (LLM), MCA data (LLM), headache incidence (McNemar) and powercalculation:

install.packages("lmerTest")

install.packages("bayesforecast")

install.packages("lmtest")

library(lme4)

library(tidyverse)

library(lmerTest)

library(nlme)

library(ggpubr)

library(lme4)

library(ggplot2)

library(nlme)

library(ggpubr)

library(bayesforecast)

library(lmtest)

library(readxl)

MCA_long_data <- read_excel("… MCA long data.xlsx")

STA_long_data <- read_excel("… STA long data.xlsx")

STA_long_data_90 <- read_excel("… STA long data 90.xlsx")

STA_long_data_120 <- read_excel("… STA long data 120.xlsx")

#specify data used

#dataset=STA_long_data_test

dataset=STA_long_data

dataset$Time <- as.numeric(as.character(dataset$Time)) # Convert to numeric

#sensitivityanalysis

model_ar1 <- lme(Measure ~ Time * Intervention,

random = ~ 1 | ID/Intervention,

correlation = corCAR1(value = 0.5, form = ~ Time | ID/Intervention),

data = dataset)

plot(ACF(model_ar1, resType="normalized"))

summary(model_ar1)

qqnorm(residuals(model_ar1))

# Augmented Dickey-Fuller Test Unit Root Test #

library(urca)

adf_test <- ur.df(dataset$Measure, type = "drift", lags = 1)

summary(adf_test)

#stationarity of data

install.packages("lmerTest")

library(readxl)

library(lme4)

library(tidyverse)

library(lmerTest)

MCA_long_data <- read_excel("… MCA long data.xlsx")

STA_long_data <- read_excel("… STA long data.xlsx")

STA_long_data_90 <- read_excel("… STA long data 90.xlsx")

STA_long_data_120 <- read_excel("… STA long data 120.xlsx")

#specify data used

dataset=STA_long_data

#NON-TRANSFORM MODEL

model <- lmer(Measure ~ Intervention * Time + (1 | ID), data = dataset)

summary(model)

#Linearity?

model <- lmer(Measure ~ Intervention * Time + (1 | ID), data = dataset)

residuals <- resid(model)

fitted_values <- fitted(model)

plot(fitted_values, residuals)

abline(h = 0, col = "red")

#Homodescasticity and normality?

qqPlot(residuals)

#LOG TRANSFORMED MODEL (MEASURE)

model_transformed <- lmer(log(Measure) ~ Intervention * Time + (1 | ID), data = dataset)

summary(model_transformed)

#Linearity?

residuals <- resid(model_transformed)

fitted_values <- fitted(model_transformed)

plot(fitted_values, residuals)

abline(h = 0, col = "red")

#Homodescasticity and normality?

qqPlot(residuals)

#Powercalculation

power.t.test(n=NULL, delta = 0.51, sd = 0.201, sig.level = 0.05, power = 0.9, type = "two.sample", alternative = "two.sided")

     Two-sample t test power calculation

              n = 4.482573

          delta = 0.51

             sd = 0.201

      sig.level = 0.05

          power = 0.9

    alternative = two.sided

NOTE: n is number in *each* group

Analysis performed in GraphPad Prism, using the function ‘Wilcoxon signed-rank test’ based on the area under the curve (‘AUC’ function), calculated with the trapezium rule, for mean heart rate (HR) from baseline to 4 hours, mean arterial pressure (MAP) from baseline to 4 hours and headache intensity from baseline to 12 hours.


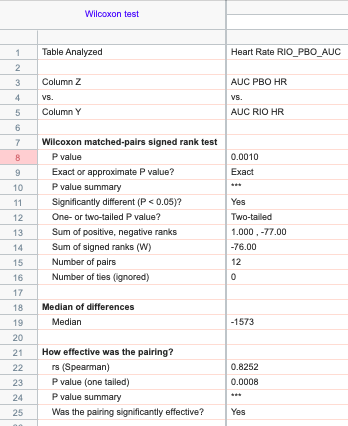

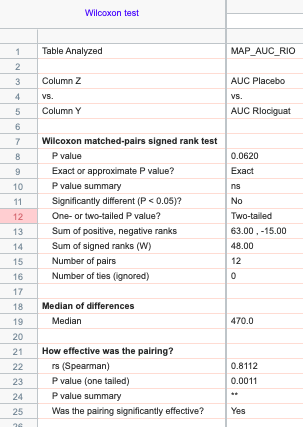

Supplement: fcaf436_Supplementary_Data [file fcaf436_supplementary_data.docx]
